# From metabolome to phenotype: GC-MS metabolomics of developing mutant barley seeds reveals effects of growth, temperature and genotype

**DOI:** 10.1038/s41598-017-08129-0

**Published:** 2017-08-15

**Authors:** Bekzod Khakimov, Morten Arendt Rasmussen, Rubini Maya Kannangara, Birthe Møller Jespersen, Lars Munck, Søren Balling Engelsen

**Affiliations:** 1 Department of Food Science, University of Copenhagen, Rolighedsvej 26, Frederiksberg, DK-1958 Denmark; 2 Department of Plant and Environmental Sciences, Copenhagen Plant Science Center, University of Copenhagen, Thorvaldsensvej 40, Frederiksberg, DK-1871 Denmark; 3 Copenhagen Prospective Studies on Asthma in Childhood, Faculty of Health and Medical Sciences, University of Copenhagen & Danish Pediatric Asthma Center, Gentofte Hospital, Copenhagen, Denmark

## Abstract

The development of crop varieties tolerant to growth temperature fluctuations and improved nutritional value is crucial due to climate change and global population growth. This study investigated the metabolite patterns of developing barley seed as a function of genotype and growth temperature for ideal vegetable protein production and for augmented β-glucan production. Seeds from three barley lines (Bomi, *lys3.a* and *lys5.f*) were sampled eight times during grain filling and analysed for metabolites using gas chromatography-mass spectrometry (GC-MS). The *lys3.a* mutation disrupts a regulator gene, causing an increase in proteins rich in the essential amino acid lysine, while *lys5.f* carries a mutation in an ADP-glucose transporter gene leading to a significant increase in production of mixed-linkage β-glucan at the expense of α-glucan. Unique metabolic patterns associated with the tricarboxylic acid cycle, shikimate-phenylpropanoid pathway, mevalonate, lipid and carbohydrate metabolism were observed for the barley mutants, whereas growth temperature primarily affected shikimate-phenylpropanoid and lipid metabolism. The study applied recently developed GC-MS metabolomics methods and demonstrated their successful application to link genetic and environmental factors with the seed phenotype of unique and agro-economically important barley models for optimal vegetable protein and dietary fibre production.

## Introduction

One of the most significant global problems in the 21^st^ century is continuous climate change, which is threatening sustainable crop production for the increasing global population^1–3^. The development through breeding and genetic engineering of crop varieties capable of adapting to new climate conditions is crucial, in order to increase yield while keeping the environmental footprint to a minimum^4, 5^. To date, several crops, including barley, have been bred for improved quality traits (QTs) such as high yield and resistance to biotic and abiotic stresses^6, 7^. Genetic modification (GM) of crops is typically performed to improve one or more desired QTs and today GM crops, including maize and rice, are permitted to be grown commercially in the US and EU.

Environmental and genetic modifications cause perturbations in the proteome and metabolome of crop plants^8, 9^. Metabolomics is becoming one of the most widely used approaches to evaluate changes in crop plants caused by GM and their possible consequences for human health and the environment^10–12^. In the past two decades, metabolomics has been established as a powerful tool within plant science to reveal the biochemical and genetic background to changes in plant behaviour as a function of GM, breeding and stresses such as drought, salinity, viruses and soil nutrition deficiency^13–16^.

This study investigated the simultaneous impacts of multiple factors, including specific genetic modifications, temperature and growth effects and their interaction effects, using gas chromatography-mass spectrometry (GC-MS) metabolomics in an experimental barley mutant model. The study involved three genotypes, the mother line (Bomi) and two near-isogenic mutants derived from Bomi, the high lysine mutant *lys3.a* (Risø 1508)^17^ with nearly ideal protein, and a high mixed-linkage β-glucan, low starch mutant *lys5.f* (Risø 13)^18^ with an ideal dietary fibre composition for human consumption.

Bomi was created as a unique barley variety in Denmark in 1969 by crossing the Bonus and Minerva varieties. The first high lysine gene, *lys*, was discovered in the Hiproly variety from Ethiopia after screening nearly 2,000 varieties from the world barley collection^19^. The high lysine mutant *lys3.a* was obtained using ϒ-rays and ethyleneimine-based GM of Bomi^17^. Among the barley mutants generated in that study, *lys3.a* was found to be exceptionally attractive, with the highest lysine to protein ratio. The relative concentration of lysine in relation to total protein concentration is elevated by 40% in *lys3.a* compared with the mother line Bomi. Shrunken seed endosperm is associated with the pleiotropic effects of the *lys3.a* gene^20^, which is located on chromosome 7^21^. Apart from showing a drastic change in protein composition, the *lys3.a* mutant also contains higher amounts of free amino acids and sugars^22^. The *lys3.a* gene is most likely found in the cluster of hordein genes^23, 24^. In the *lys5.f* mutant, generated by ethyleneimine-based GM of Bomi^18^, the content of α-glucan is decreased (from 49 to 30%), while the mixed-linkage β-glucan content is increased (from 7 to 20%) compared with Bomi^25^. The *lys5.f* phenotype is caused by a mutation in the *Hν.NST1* gene, located on chromosome 6. This gene encodes an ADP-glucose (ADP-Glc) transporter protein in barley^26^. Due to the *lys5.f* mutation, the ADP-Glc transporter protein is impaired, leading to a reduction in starch content^26^.

**Figure 1:**
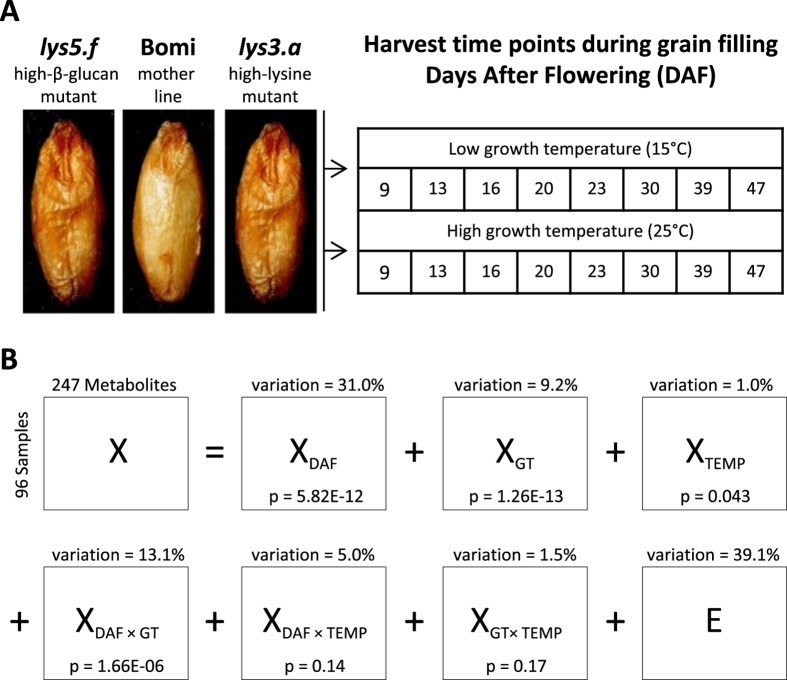
Overview of the study design and variance contribution in metabolomics data. (**A**) Three barley genotypes (GT): high lysine mutant *lys3.a*, low starch high β-glucan mutant *lys5.f* and the mother line Bomi, were grown under the same conditions in the greenhouse at two different growth temperatures (TEMP) and sampled at eight time points during days after flowering (DAF). The final metabolomics data included 96 samples (3GT × 2TEMP × 8DAF × 2REPLICATES) and 247 variables after processing of the untargeted raw GC-MS data using PARAFAC2 (Supplementary Figure S1 and Table S1). (**B**) ANOVA-simultaneous component analysis (ASCA)-based decomposition of DAF, GT and TEMP and their two-factor interaction effects as the overall variance contribution of effects across all metabolites. The null hypothesis (H0) was checked for each main and interaction effect using sum of squares of effect matrices, e.g. **X**_**DAF**_, and p-values were assessed by permutation test (5,000 permutations).

In this study, the seed metabolome of all three genotypes, Bomi, *lys3.a* and *lys5.f*, grown at two different temperatures, was measured during grain filling (Fig. 1A). The multidisciplinary metabolomics approach applied, together with an advanced multivariate data mining technique exploiting the experimental design^27^, was intended to uncover metabolic alterations in seeds in response to GM, temperature, grain filling and their interactions and to provide novel insights into biochemical pathways in the barley mutant model.

## Results

The final metabolomics data included 247 variables after processing of the raw GC-MS data using PARAllel FACtor Analysis2 (PARAFAC2)^28^ (Supplementary Figure S1). A total of 104 metabolites were identified at level 2, based on Metabolomics Standards Initiative^29^, and included organic acids, aldehydes, alcohols, polyols, phenolic compounds, flavonoids, fatty acids, carbohydrates and other chemical classes (Supplementary Table S1). The balanced design of the study allowed unambiguous partitioning of variances originating from the main effects, genotype (GT), temperature (TEMP), days after flowering (DAF) and their two-factor interaction effects, using ANOVA-simultaneous component analysis (ASCA)^27^. A total of six systematic terms (three main effects (**X**_**GT**_, **X**_**DAF**_, **X**_**TEMP**_) and three two-factor interaction effects (**X**_**GT** × **TEMP**_, **X**_**DAF** × **TEMP**_, **X**_**GT** × **DAF**_)) and a single random effect (**E**) were evaluated (Fig. 1B). All main effects and the interaction effect between GT and DAF were significant, and further data analysis and interpretation were performed on the ASCA-based separated effect matrices, **X**_**GT**_, **X**_**DAF**_, **X**_**TEMP**_ and **X**_**GT** × **DAF**_. The highest systematic variation (31.0%), which derived from the DAF effect, revealed a substantial change in the seed metabolome during grain filling. The second largest variation (13.1%) was due to GT-specific DAF effects, with the GT effect contributing 9.2% of the total variation. Although growth temperature gave the smallest variation (1.0%), it was still statistically significant (p = 0.04). The two-factor interaction terms **X**_**DAF** × **TEMP**_ and **X**_**GT** × **DAF**_ were not significant. The residual matrix, **E**, explained the variation (39.1%) derived from individual differences between plant biological replicates, experimental errors and other variations not related to the study design.

### Alteration of the barley seed metabolome in mutants

**Figure 2:**
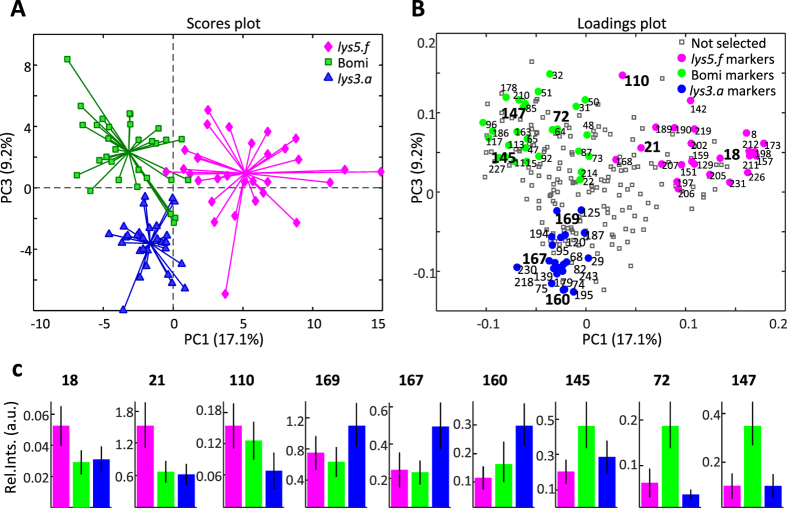
Principal component analysis (PCA) of the ANOVA-simultaneous component analysis (ASCA)-based genotype effect separated matrix, **X**_**GT**_. (**A**) Scores plot of the PCA model developed on **X**_**GT**_. (**B**) The loadings plot of the corresponding PCA model highlights the most discriminative metabolite markers for each genotype, selected by the corresponding PLS-DA models using a variable selection approach. (**C**) Relative mean concentrations of some metabolite markers, calculated from the raw data, for *lys5.f* (18: maltol, 21: 3-methyl-2-furoate, 110: α-methyl-4-hydroxymandelic acid, methyl ester), Bomi (169: sinapinic acid, methyl ester, 167: isoferulic acid, 160: 2-hydroxysebacic acid) and *lys3.a* (145: gallic acid, 72: 2-ketoglutaric acid, 147: 4-hydroxymandelic acid, ethyl ester).

Principal component analysis (PCA)^30^ of the raw metabolite data revealed only partial separation of the barley genotypes, due to confounding effects such as DAF masking the GT effect (data not shown). In the PCA analysis of the ASCA-based separate GT effect, **X**_**GT**_, which was free of confounding effects, all three genotypes were clearly discriminated (Fig. 2A). *lys5.f* was separated from Bomi and *lys3.a* along principal component 1 (PC1) explaining the major variation (17.1%), while *lys3.a* was discriminated from Bomi along PC3 (9.25%). This suggests a larger metabolome-wide alteration in *lys5.f* than in *lys3.a* compared with Bomi. The detailed metabolic alterations behind these discriminations were studied by partial least squares-discriminant analysis (PLS-DA)^31^ (Fig. 2B). All PLS-DA models developed to classify GTs were validated using independent test set samples and were found to give a 0–5% misclassification rate, while the area under the receiver operating characteristics curve (AUC) was at least 0.95 (Supplementary Figure S2). PLS-DA based variable selection allowed identification of 72 GT effect metabolite markers, 24 for *lys5.f*, 27 for Bomi and 21 for *lys3.a*. A total of 62 of those biomarkers were also found to be significant in the univariate analysis (p < 0.05). The univariate test revealed 41 additional significant variables that could account for the discrimination of at least two genotypes (Supplementary Table S1).

The most discriminative markers for Bomi were 3-hydroxyhepnateoate, 2-ketoglutaric acid, succinic acid, malic acid, 3-hydroxy-3-methylglutaric acid, 4-hydroxybutyric acid, gallic acid, 4-hydroxymandeleic acid, methyl ester of 3,4-dihydroxy-benzoic acid and protocatechuic acid. These metabolites, along with others (Supplementary Table S1), were reduced in their relative concentrations in the mutants compared with Bomi, as also confirmed by the raw metabolomics data (Fig. 2C). The high β-glucan low starch mutant, *lys5.f*, showed relatively higher levels of 3-methyl-2-furoate, maltol and α-methyl-4-hydroxymandelic acid methyl ester compared with Bomi and *lys3.a*. The most discriminative markers identified for the high-lysine mutant, *lys3.a*, were threonic acid, dihydroxyacetone, methyl ester of sinapinic acid, isoferulic acid, 2-hydroxyheptanoate, 2-hydroxysebacic acid and dimethyl ester of *threo*-9,10-dihydroxy-octadecanedioic acid.

In order to evaluate metabolite classes and their contributions to the separation of the three barley genotypes, PCA analysis of the genotype effect separated matrix, **X**_**GT**_, using only identified metabolites, was performed. It showed partial separation of the *lys3.a* genotype due to the higher fatty acid content (data not shown). This finding is in agreement with previous results reported for the same genotypes, which revealed an elevated level of total lipids in *lys3.a* ^32^. Earlier studies have shown that the high mixed-linkage β-glucan content (at the expense of starch) in developing seeds of *lys5.f* results in an up to 10% increase in the water content compared with Bomi^33^. Similarly, the *lys3.a* mutant contains a higher level of hydrophilic high lysine containing proteins at the expense of hydrophobic storage proteins^32^, which increases the water content and water activity during epigenesis. The differences in water binding and water activity between the mutants and Bomi are likely to influence the pleiotropy of the seed metabolome, as found in this study. These differences in the barley endosperm metabolome and their possible relations to altered biosynthetic pathways and related pleiotropic effects in the plant are addressed in later sections of this paper.

### Effects of growth temperature

**Figure 3:**
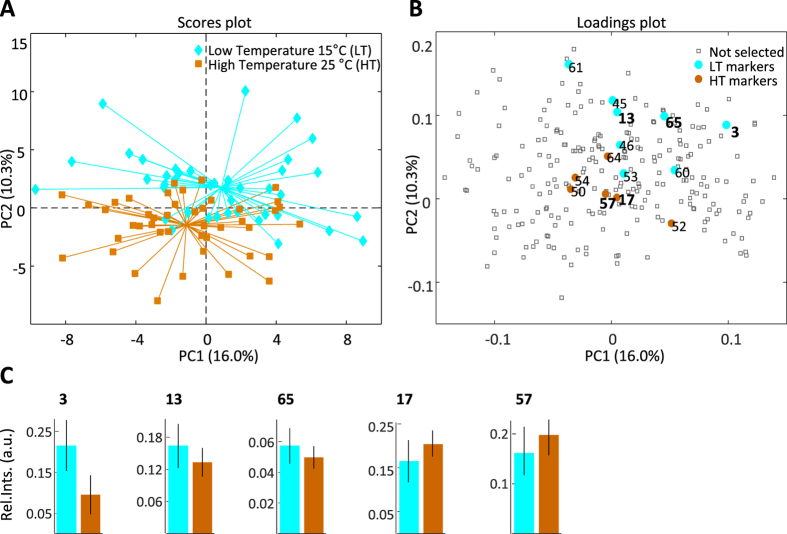
Principal component analysis (PCA) of the ANOVA-simultaneous component analysis (ASCA)-based growth temperature effect separated matrix, **X**_**TEMP**_. (**A**) Scores plot of the PCA model developed on **X**_**TEMP**_. (**B**) Loadings plot of the same PCA model. Metabolite markers for the temperature effect were selected from the PLS-DA models using a variable selection approach. (**C**) Relative mean concentrations of some metabolite markers, calculated from the raw data, for the low growth temperature (15 °C) (3: hepta-2,4-dienoic acid, methyl ester, 13: benzoic acid, 65: 3-hydroxybenzoic acid) and the high growth temperature (25 °C) (17: glycerol, 57: p-hydroxyphenylactic acid, methyl ester).

The effects of growth temperature were smaller than those of DAF and GT (Fig. 1), and a PCA analysis was not sufficient to reveal the effect. However, the effect became clearer after removing the confounding factors using ASCA. A partial separation of barley grown at low (LT) and high (HT) temperature could be detected in the first two PCs, explaining 26% of the variation, when **X**_**TEMP**_ was analysed by PCA (Fig. 3A). A total of 14 most discriminative growth temperature metabolite markers were identified by PLS-DA variable selection and included nine LT and five HT markers (Fig. 3B). The validated PLS-DA model depicted high classification power, with an error of 3% and an AUC of 0.90 (Supplementary Figure S3).

Among growth temperature-related markers, four of the LT markers were identified as benzoic acid, pyroglutamic acid, 3-hydroxybenzoic acid and methyl ester of hepta-2,4-dienoic acid. The HT barley plants contained relatively higher levels of glycerol and 4-hydroxyphenylacetic acid methyl ester. A univariate test revealed five variables that were significant for the growth temperature (Supplementary Table S1), but only one, methyl ester of hepta-2,4-dienoic acid, matched the multivariate biomarkers. Due to the relatively smaller effect of the growth temperature, differences in mean concentrations of metabolites were minor between the two groups (Fig. 3C). The fact that the genotype-temperature interaction effect (**X**_**GT** × **TEMP**_ **)** was not significant suggests that the influence of growth temperature was similar for all three genotypes (Fig. 1B). However, although the interaction between temperature and DAF (**X**_**TEMP** × **DAF**_ **)** was not significant, temperature-related variations in identified metabolite markers were more pronounced at the earlier DAF.

### The developing metabolome during grain filling: common and GT-specific DAF effects

**Figure 4:**
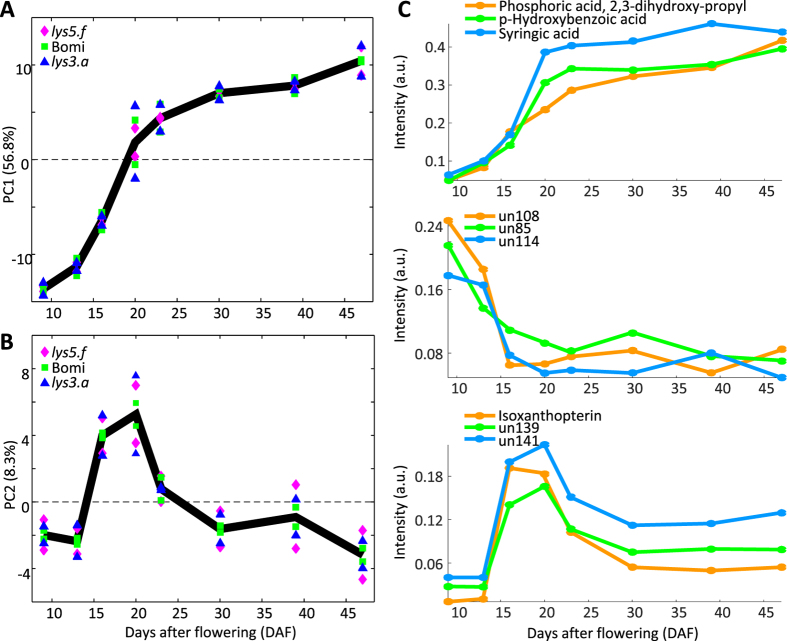
Development of the barley seed metabolome during grain filling. Principal component analysis (PCA) of the ANOVA-simultaneous component analysis (ASCA)-based days after flowering (DAF) effect separated matrix, **X**_**DAF**_, revealed three main trends over which relative concentrations of metabolites changed during grain filling. (**A**) Principal component (PC)1 explained more than half of the total variation and revealed two trends, accumulation and depletion. (**B**) PC2 of the same model explained 8% variation and revealed an accumulation followed by depletion trend. (**C**) Relative mean concentrations, calculated from the raw data, showing some metabolite examples that follow one of the three developmental trends.

During post-anthesis grain filling, the barley seed metabolome gradually changed, which was observed as the greatest variation, and revealed common **DAF (X**_**DAF**_), and GT-specific DAF (**X**_**DAF** × **GT**_) effects (Fig. 1B). PCA of **X**_**DAF**_ revealed metabolite accumulation, depletion and accumulation, followed by depletion trends (Fig. 4). The latter trend was in agreement with a similar proteome effect reported in the same barley mutants^32^. A PLS regression model was developed on **X**_**DAF**_, enabling prediction of the grain filling stages with high accuracy (r^2^ = 0.83, root mean square error of prediction (RMSEP) = 5.25 DAF) (Supplementary Figure S4). Metabolites that explained the common DAF effect in the PCA analysis of **X**_**DAF**_ were also found to be the most predictive variables in the PLS regression model. Moreover, the majority of these metabolites were also significant for the DAF effect in the univariate tests (Supplementary Table S1). Based on PCA and PLS analyses, 136 metabolites were found to follow an accumulation trend, 28 a depletion trend and 10 an accumulation followed by depletion trend during grain filling (Supplementary Table S1).

Likewise, for **X**_**DAF**_ the PCA model of **X**_**DAF** × **GT**_ revealed the same three major trends in the developing seed metabolome (Supplementary Figure S4). The accumulation and depletion trends were captured in PC1 (38% variation), where the difference in metabolite levels between genotypes became clearer after 25 DAF. PC2 (11% variation) depicted the accumulation followed by depletion trend and revealed a distinct difference between *lys5.f* and the other two genotypes. However, due to the rotational freedom of the PCA model, the PC2 of the **X**_**DAF** × **GT**_ was inversed. The genotype-specific DAF effects were mostly dominated by organic acids.

### Correlation of metabolites during the grain filling

**Figure 5:**
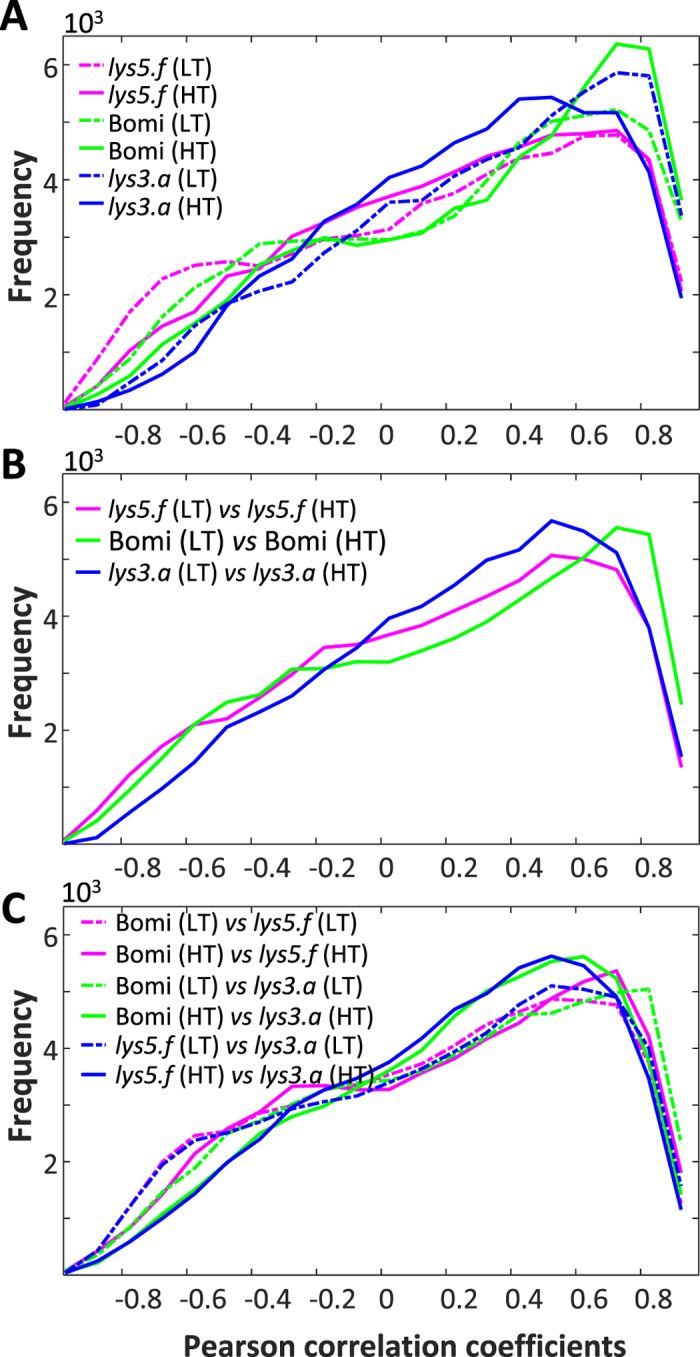
Distribution of Pearson correlation coefficients among metabolites during grain filling. (**A**) Distribution based on the correlation coefficients among metabolites within each genotype (GT) grown at two different temperatures. (**B**) Distribution based on the correlation coefficients among metabolites between the same genotype, but grown under different temperatures. (**C**) Distribution based on the correlation coefficients among metabolites between different genotypes, but grown under the same temperature. Although the **X**_**GT**_ × **X**_**TEMP**_ interaction effect was not significant (Fig. 1), a higher number of positive correlations was observed between Bomi and *lys3.a* at LT than between Bomi and *lys5.f*. This is in agreement with ANOVA-simultaneous component analysis (ASCA) of the GT effect, which showed a greater difference between Bomi and *lys5.f* than between Bomi and *lys3.a*. However, at the high temperature (HT) growth condition these correlations were no longer preserved, but the number of positive correlations was slightly increased between Bomi and *lys5.f*. Heatmaps of the corresponding correlation coefficient matrices and the number of significantly positive and negative correlations in those correlation coefficient matrices are illustrated in Supplementary Figs. S5–S7.

Pearson correlation analysis of metabolites over eight developmental stages within each genotype and growth temperature revealed significant positive and negative correlations between metabolites that may be related to direct or indirect relationships in biosynthetic pathways. Heatmaps of metabolite correlations during growth were unique to genotypes and growth temperatures, and can be considered a fingerprint of the efficacy and/or stress level of the plant (Supplementary Figure S5). Unsurprisingly, the majority of significant correlations were positive, due to the metabolite build-up during grain filling. Significant inverse relationships of metabolite biosynthesis were consistently more pronounced in the LT barley genotypes. At both growth temperatures, the number of negative correlations was increased in *lys5.f* and decreased in *lys3.a* mutants compared with Bomi. However, positive correlation trends among metabolites were more temperature-dependent. Positive correlations among the HT plants were highest in Bomi, while for LT plants *lys3.a* had the highest number. The numbers of positive correlations were almost same in *lys5.f* grown at both temperatures, while for Bomi the number increased and for *lys3.a* it decreased at high compared with low growth temperature. The distributions of the Pearson correlation coefficients were also unique for each genotype and growth temperature (Fig. 5A). These results show that both GM and growth temperature alter the developing seed metabolome and that possible deregulation of metabolic synergy may occur in the mutants.

The Pearson correlation analysis of same genotypes revealed a change in seed metabolome dynamics over DAF when plants were grown at different temperatures (Fig. 5B and Supplementary Figure S6). However, this change was less extensive in Bomi than in the mutants and Bomi showed the highest number of significant positive correlations. Interestingly, the number of significant negative correlations increased for *lys5.f* and decreased for *lys3.a* compared with Bomi, as was also observed for within-genotype metabolite correlations (Fig. 5A). It can be assumed that the higher number of positive correlations in Bomi suggests more concerted metabolic development than in the mutants and thus also less influenced by the temperature change.

Analysis of correlations of metabolites between different genotypes, grown at the same temperature, showed that the highest number of positive correlations was between Bomi and *lys3.a* at LT (Fig. 5C and Supplementary Figure S7). The number of positive correlations was slightly higher between *lys3.a* and *lys5.f* than between Bomi and *lys5.f* at LT. In the HT conditions, the numbers of positive correlations between *lys3.a* and Bomi, and between *lys5.f* and Bomi, were similar, whereas there was a much lower number of positive correlations between the two mutants under the same HT conditions. The latter result suggests that the mutants are more similar to the mother line Bomi than to each other. The numbers of negative correlations were always higher in genotypes grown at LT than in genotypes grown at HT. This might be related to the developing seed metabolome being more controlled during LT growth, while during HT growth the metabolic equilibrium becomes slightly more chaotic.

## Discussion

Global top-down studies using near-infrared spectroscopy have shown that the *lys5.f* and *lys3.a* barley mutants display specific chemical patterns during seed development that are highly reproducible when the mutants are grown in a controlled environment^25, 32^. Initial protein synthesis is different for each mutant genotype and for the mother line, Bomi. It is faster in *lys5.f*, followed by *Bomi* and *lys3.a*, although at 23 DAF the protein synthesis rate in *lys3.a* accelerates and leads to the highest protein level^32^. These differential protein synthesis kinetics at different grain filling stages can of course be expected to influence the seed metabolome too. In the following, the details of the barley seed metabolome as a function of the barley mutant model are discussed.

**Figure 6:**
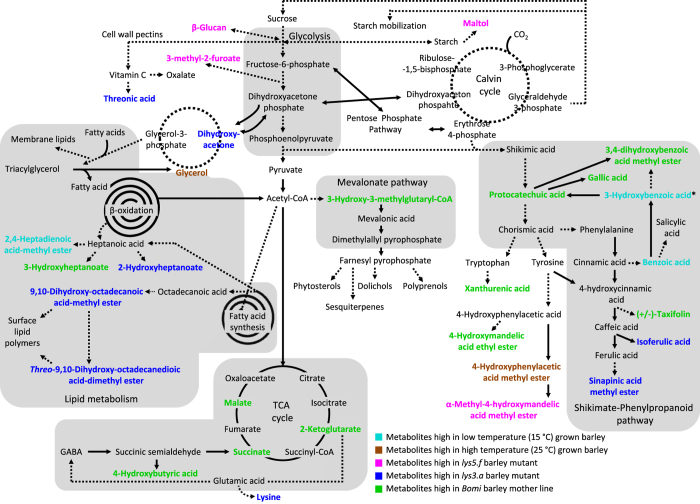
General overview of the major metabolic pathways in the barley plant and statistically significant metabolite markers for the growth temperature and genotype effects. Metabolite markers for the low (LT) and high (HT) temperature growth conditions are highlighted in cyan and brown, respectively. Metabolite markers for the genotype are highlighted in magenta, blue and green for *lys5.f*, *lys3.a* and Bomi, respectively. Solid arrows represent single enzymatic steps prior to metabolite biosynthesis, while dotted arrows show multi-enzymatic steps. P-values from the ANOVA test and fold changes in relative concentrations of markers are summarised in Supplementary Table S1.

Introduction of the high-lysine *lys3.a* gene into barley causes a decrease in hordein, a prolamin glycoprotein, with a concomitant increase in lysine-rich, water-soluble proteins in the endosperm^17^. In contrast, the *lys5.f gene* changes the carbohydrate composition of the barley endosperm by reducing starch and increasing the mixed-linkage β-glucan content. Alterations in protein and carbohydrate composition are not the only metabolic changes caused by the induced mutations. In fact, multiple metabolic pathways are affected by the mutations, which cause metabolic changes that are more pronounced in the tricarboxylic acid (TCA) cycle, where the relative levels of organic acids such as malate, succinate, 2-ketoglutarate and 4-hydroxybutyric acids are significantly reduced compared with the levels in their mother line Bomi (Fig. 6). In addition, gallic acid, protocatechuic acid, taxifolin and the methyl ester of 3,4-dihydroxybenzoic acid derived from the shikimate-phenylpropanoid pathway are significantly reduced in the mutants. The reduction of TCA cycle metabolites in *lys3.a* may be due to increased lysine synthesis in this mutant (Fig. 6). This observation is consistent with data previously reported for *Arabidopsis thaliana (A. thaliana)*, where a tight link between the TCA cycle and lysine metabolism has been demonstrated to occur in developing seeds^34, 35^. For example, a reduced level of citrate, 2-ketoglutarate and fumarate has been reported in seeds that are genetically engineered to accumulate high levels of lysine during development compared with wild-type *A. thaliana* seeds.

Several intermediates of the shikimate-phenylpropanoid pathway are altered in *lys3.a* compared with Bomi. Interestingly, there is a marked elevation of isoferulic acid and sinapinic acid methyl ester in *lys3.a*, together with a reduction in the protocatechuic acid content. This could indicate that the mutant is able to rapidly convert protocatechuic acid into chorismic acid and other downstream biosynthetic intermediates that lead to the production of isoferulic acid and sinapinic acid methyl ester.

In addition, three intermediates of lipid metabolism, namely 2-hydroxyheptanoate, 9,10-dihydroxy-octadecanoic acid methyl ester and dimethyl ester of threo-9,10-dihydroxy-octadecanedioic acid, are increased in the high-lysine mutant *lys3.a* compared with *lys5.f* and Bomi. The dihydroxyacetone level is also upregulated in *lys3.a* and this might explain the increased flux through the lipid metabolic pathway, as dihydroxyacetone is an early precursor of triacylglycerol storage lipids (Fig. 6). Another possible explanation could be that as the *lys3.a* mutant accumulates high amounts of lysine and other free amino acids, it has to cope with this surplus. One option is to increase amino acid incorporation into lysine-rich proteins, which indeed occurs in the *lys3.a* mutant^17, 36^. Alternatively, the plant may increase lysine catabolism into acetyl-CoA, thus giving rise to more precursors for fatty acid synthesis and resulting in increased formation of 2-hydroxyheptanoate, 9,10-dihydroxy-octadecanoic acid methyl ester and dimethyl ester of threo-9,10-dihydroxy-octadecanedioic acid (Fig. 6).

Less localised metabolites are increased in the high β-glucan mutant, *lys5.f*, compared with Bomi and *lys3.a*. Among these, maltol and 3-methyl-2-furoate, which are related to carbohydrate metabolism, could potentially be linked to the altered content of starch and mixed-linkage β-glucan detected in *lys5.f* ^25^. An elevated level of the methyl ester of α-methyl-4-hydroxymandelic acid, together with a reduced level of xanthurenic acid in *lys5.f*, may indicate that there is a diverted metabolic flux of protocatechuic acid in this mutant compared with Bomi (Fig. 6). It can be assumed that the observed changes in metabolite patterns found in *lys3.a* and in *lys5.f*, compared with Bomi, are a direct consequence of either altered expression of genes or altered regulation of enzymes associated with the metabolic pathways affected. Patron *et al*.^26^ isolated three independent *lys5* mutants that were all characterised as having a low-starch phenotype, due to their inability to synthesise starch at normal rates from ADP-Glc, and which were found to contain a lesion in the *Hv.NST1/lys5* gene cluster, which encodes a plastidial ADP-Glc transporter. Thus, it can be inferred that the low-starch phenotype observed in *lys5* mutants is caused by this specific lesion in ADP-Glc transportation. The mutation in an ADP-Glc transporter, *Hv.NST1/lys5* gene cluster, may also play a key role for the diverse metabolic differences induced in the mutants by either indirectly up- or down-regulating the biosynthesis of different classes of metabolites. This hypothesis is supported by the fact that the closest known homologous protein to barley Hv.NST1 is a maize membrane-bound transporter, brittle-1^34^. Brittle-1 belongs to a family of transporters that are mostly are located in the mitochondrial outer envelope and are involved in transportation of many different kinds of metabolites^37^.

Temperature affects the growth of barley at all stages from germination to grain filling and determines the yield^38^. The lower growth temperature of 15 °C investigated in this study is considered optimal for barley, while under high temperature (25 °C) the yield, including starch and protein content, decreases as a result of the shortened grain filling duration, as also observed in this study. The high temperature employed in this study is below the heat shock temperature (>30 °C) at which barley encounters reduced grain filling rates and irreversible shock leading to significant yield losses. The relatively smaller effect of growth temperature, compared with genotype, can be explained by the fact that 25 °C is still within the plateau of the thermal kinetic window of barley^39^. The relative levels of two metabolites of the shikimate-phenylpropanoid pathway, benzoic acid and 3-hydroxybenzoic acid, and the methyl ester of 2,4-heptadienoic acid, which is part of lipid metabolism, were consistently increased in all barley genotypes grown under LT conditions compared with the corresponding genotypes grown under HT conditions (Fig. 6). The elevation of benzoic acid under LT conditions is not surprising, since it has been shown that in *A. thaliana*, cold stress induces production of the phytohormone salicylic acid (2-hydroxybenzoic acid)^40^. Benzoic acid is a direct precursor of salicylic acid and thus it is plausible that barley lines grown under LT conditions are seeking to increase the level of this phytohormone in order to adapt to cold stress. The increased level of pyroglutamic acid in LT barley plants suggests that cold stress upregulates glutamate synthesis, as also shown in a metabolomics study investigating an effect of cold stress on *Arabidopsis lyrata* ssp*. petraea* ^41^.

In contrast, the levels of glycerol and methyl ester of 4-hydroxyphenyllactic acid were increased in all genotypes grown under HT conditions compared with under LT conditions. In particular, the changes in glycerol content in response to growth temperature are interesting and may be linked to alterations in storage triacylglycerol lipids and membrane lipids. In sunflower (*Helianthus annuus* L.) seeds, it has been shown that high temperatures can trigger an increase in triacylglycerols, diacylglycerols and membrane phospholipids, all of which are derived from glycerol (Fig. 6)^42^.

The Pearson correlation analysis of metabolites over DAF, between the same and different genotypes, showed that the numbers of significant negative and positive correlations were higher under LT compared with HT conditions. This may indicate that both GM and growth temperature alter the developing seed metabolome and that possible deregulation of metabolic synergy may occur in the mutants. The higher numbers of significant positive and negative correlations between the metabolites of the mother line Bomi grown under LT and HT compared with the mutants suggest that the effect of growth temperature was smaller in Bomi than in the mutants (Fig. 5B) and may indicate greater plasticity of Bomi to temperature change.

During grain filling in barley seeds, several physiological changes occur that most likely affect not only the metabolite composition of the seeds, but also the enzymatic and gene expression profiles. In the present study, we observed that during grain filling of the seed, 136 metabolites accumulated, 28 metabolites depleted and 10 metabolites accumulated and then depleted. Overall, the class of flavonoids and phenolic compounds identified showed the highest degree of increase during grain filling. All flavonoids measured were shown to accumulate and this was also demonstrated for 84% of the phenolic compounds identified (Supplementary Table S1). The increase in flavonoids in particular is consistent with previous observations in rice, with a significant increase in this class of metabolites occurring in four different rice cultivars during the reserve accumulation stage of the grain^43^.

This study established a link between genetic and environmental factors and the phenotype, the metabolome, in barley mutant models. The metabolomics approach combined with advanced chemometrics showed mutant-specific and growth temperature-dependent metabolic effects on different biosynthetic pathways. The barley mutant model studied here possesses a near-optimal protein (rich in essential amino acids) and dietary fibre (mixed-linkage β-glucan) composition. Moreover, barley can be relatively easily adapted to climate change and thus a better understanding of the link between genome and phenome is of paramount interest for sustainable production of nutritious protein and dietary fibre. Development of crops with an ideal protein composition will lower the environmental footprint of animal production by replacing synthetic and non-locally produced protein supplements. This may further reduce nitrogen pollution and increase animal welfare. Increased production of mixed-linkage β-glucan in barley has great potential to promote development of new health-promoting foods. Future studies on sequencing of these barley mutants and fusion of genome, transcriptome and proteome data with metabolome data will further advance understanding of how genetic and environmental factors affect crop growth. Such studies may open up new possibilities to develop crop varieties that are tolerant to climate change and have improved nutritional value and high yield, thus enabling existing agricultural land to be used more efficiently.

## Materials And Methods

### Plant materials and study design

Seeds of the barley lines Bomi, *lys3.a* and *lys5.f* were grown under the same conditions using a semi-field pot experiment at low (15 °C; LT) and high (25 °C; HT) temperature. Details of the experiment, including growth, harvest, chemical analysis and genetic modifications of Bomi, have been reported previously^17, 18, 44^. The spikes on the main and the first side tillers were harvested at eight different time points during grain filling: 9, 13, 16, 20, 23, 30, 39 and 47 days after flowering (DAF). Two biological replicates were sampled at each time point. Thus, a total of 96 whole-grain samples: 3 genotypes (GT) × 8 sampling days after flowering (DAF) × 2 different temperatures (TEMP: LT and HT) × 2 biological replicates, were lyophilised and stored at −20 °C until GC-MS analysis (Fig. 1A).

### Metabolite analysis

A 50 mg portion of lyophilised whole-grain flour was soaked in 600 µL 85% methanol and vortexed for 20 s at 3000 rpm, followed by 20 min of incubation at 30 °C using a Thermomixer (Eppendorf, USA) at 1400 rpm. After 3 min of centrifugation at 16.000 g, the supernatant was transferred to a fresh 2-mL plastic Eppendorf tube and the remaining flour sample was extracted once more using the same procedure. The combined supernatants were completely dried under nitrogen gas at 40 °C and hydrolysed using 240 µL of 6 M hydrochloric acid at 96 °C for 1 hour under stirring at 1400 rpm. The hydrolysed extract was transferred to a fresh 2-mL HPLC glass vial, 800 µL of diethyl ether were added and the mixture was vortexed prior to metabolite extraction. This ether-based extraction was repeated twice. The combined ether fraction was dried using nitrogen gas and re-solubilised in 200 µL 100% methanol. A 90 µL aliquot of the final extract was transferred to a 200-µL glass insert, completely dried under nitrogen flow, sealed and stored at −20 °C until GC-MS analysis (up to 72 hours). Each sample was spiked with an internal standard, 5 µL of a 0.2 mg mL^−1^ solution of ribitol. Prior to GC-MS injection, the samples were derivatised using trimethylsilyl cyanide (TMSCN)^45^. A 40 µL aliquot of TMSCN reagent was added to each sample, and the samples were then tightly sealed in GC-MS vials using silicon septum magnetic lids and incubated for 40 min at 40 °C before 1 µL of sample was injected into the cooled injection system (CIS4 port) of the GC-MS. Samples were randomised before analysis, and derivatisation and injection were fully automated using a MPS autosampler (GERSTEL, Mülheim, Germany) integrated to the GC-MS (Agilent Technologies, Glostrup, Denmark). The GC-MS consisted of an Agilent 7890A GC and an Agilent 5975C series MSD. GC separation was performed on a Phenomenex ZB 5MSi column (30 m × 250 µm × 0.25 µm). Sample injection mode was Solvent Vent at a vent pressure of 7 kPa until 0.3 min after injection and the vent flow was 100 mL min^−1^. Hydrogen was used as carrier gas, at a constant flow rate of 1.2 mL min^−1^, and initial temperature of the CIS4 port was set to 120 °C until 0.3 min, followed by heating at 5 °C s^−1^ until 320 °C and holding for 10 min. The GC oven programme was as follows: initial temperature 40 °C, equilibration time 3.0 min, heating rate 12.0 °C min^−1^, end temperature 300 °C, hold time 8.0 min and post run time 5 min at 40 °C. Mass spectra were recorded in the range 50–500 *m*/*z* with a scanning frequency of 3.2 scans s^−1^, and the MS detector was switched off during the 8.5 min of solvent delay time. The transfer line, ion source and quadrupole temperature was set to 290, 230 and 150 °C, respectively. The mass spectrometer was tuned according to the manufacturer’s recommendation using perfluorotributylamine (PFTBA).

### Data processing

The raw GC-MS data were processed by a multi-way decomposition technique, PARAFAC2^28^, in order to deconvolute metabolite peaks. PARAFAC2 allowed simultaneous deconvolution of GC-MS peaks from the whole data, including elusive peaks like co-eluted, RT-shifted and low signal-to-noise (S/N), using original three-way structure, elution time points (4800) × mass spectra (450) × samples (96) of the data^46^. In order to simplify and speed up PARAFAC2, the raw data were divided into 121 smaller intervals in the elution time dimension and each interval was modelled individually, followed by validation as previously described^46^. The PARAFAC2 models provide three important outputs for each deconvoluted peak: 1) elution profile (equivalent to total ion current); 2) spectral profile (equivalent to mass spectra of peak); 3) concentration profile (equivalent to peak area) (Supplementary Figure S1). Elution and spectral profiles assisted in metabolite identification based on retention index (RI) and electron impact-mass spectra (EI-MS) at level 1 and 2^28^, using the NIST11 database (Version 2.0, NIST, USA). The final metabolite table **X**_***(i,k)***_, based on the PARAFAC2 concentration profiles, was normalised by the area of the internal standard, **Xi**_***(i,k)***_ = **X**_***(i,k)***_ **/IS**_***(i)***_, where ***i*** and ***k*** correspond to samples and variables, respectively. Furthermore, in order to remove a closure effect derived from the starch accumulation during grain filling that causes natural dilution of metabolites in seeds, **Xi**_***(i,k)***_ was normalised by seed dry matter weight, **Seed**_***(i)***_, as **Xs**_***(i,k)***_ = **Xi**_***(i,k)***_ × **Seed**_***(i)***_. All subsequent data analyses were performed on the final metabolite data matrix, **Xs**_***(i,k)***_.

### Statistical Analysis

Univariate analysis, including one-way ANOVA, and multivariate data analysis, principal component analysis (PCA)^30^, ANOVA-simultaneous component analysis (ASCA)^27^, partial least squares regression analysis (PLS)^47^ and PLS-discriminant analysis (PLS-DA)^31^ were performed to study the metabolic effects of design factors, GT, TEMP, DAF and their interactions. Multivariate data analyses were performed on mean-centred and scaled data. The study design factors were systematically evaluated by ASCA, which partitions variances according to the design factors in a similar fashion as in one-way ANOVA, followed by exploratory analysis of the effect matrices, e.g. **X**_**DAF**_, using either PCA or simultaneous component analysis (SCA). In order to interpret only significant effects, the systematic effects were evaluated one-by-one by random permutation testing^48^ (Fig. 1B). The PLS and PLS-DA were employed to select the most relevant metabolite markers for GT, TEMP and DAF using a variable selection method based on variable importance for projection (VIP) scores and regression coefficients, as described previously^49^. Regression models were developed on effect matrices, e.g. **X**_**DAF**_, and optimised and validated using the double cross-validation procedure^50^. Pearson correlations were calculated on the raw data, where 247 metabolites were correlated over the eight DAF points either within or between genotypes, grown under the same or different temperatures. Correlation tables (247-by-247) from such analyses were used to evaluate the distributions of the Pearson correlation coefficients. All chemometrics analyses were performed in MATLAB® ver. R2015a (8.5.0.197613) using in-house algorithms.

### Data Availability Statement

Upon acceptance of the manuscript, the dataset used in this study will be made publicly available via the EBI MetaboLights metabolomics data repository and on a local server at http://www.models.life.ku.dk

## Electronic supplementary material

Supplementary Figures

Supplementary Information
