# Supplementary material for: From metabolome to phenotype: GC-MS metabolomics of developing mutant barley seeds reveals effects of growth, temperature and genotype
Source: Sci Rep. 2017 Aug 15;7:8195. doi: 10.1038/s41598-017-08129-0 (PMC5557882; doi:10.1038/s41598-017-08129-0)
Supplement: Supplementary file 1 — Supplementary Figures [file 41598_2017_8129_MOESM1_ESM.pdf]

From metabolome to phenotype: GC-MS metabolomics of developing mutant barley seeds reveals effects of growth, temperature and genotype

Bekzod Khakimov[*,1,2], Morten Arendt Rasmussen[1,3], Rubini Maya Kannangara[2], Birthe Møller Jespersen[1], Lars Munck[1], Søren Balling Engelsen[*,1]

[1]Department of Food Science, University of Copenhagen, Rolighedsvej 26, Frederiksberg DK-1958, Denmark, [2]Department of Plant and Environmental Sciences, Copenhagen Plant Science Center, University of Copenhagen, Thorvaldsensvej 40, Frederiksberg CDK-1871, Denmark, [3]Copenhagen Prospective Studies on Asthma in Childhood, Faculty of Health and Medical Sciences, University of Copenhagen & Danish Pediatric Asthma Center, Gentofte Hospital, University of Copenhagen; Denmark.

Corresponding author: Dr. Bekzod Khakimov, Email: bzo@food.ku.dk, Tel.: +45 35332974, Address: Rolighedsvej 26, 1958 Frederiksberg C, Denmark

Co-corresponding author: Prof. Søren Balling Engelsen, E-mail: se@food.ku.dk, Tel.: +45 35333205, Address: Rolighedsvej 26, 1958 Frederiksberg C, Denmark.

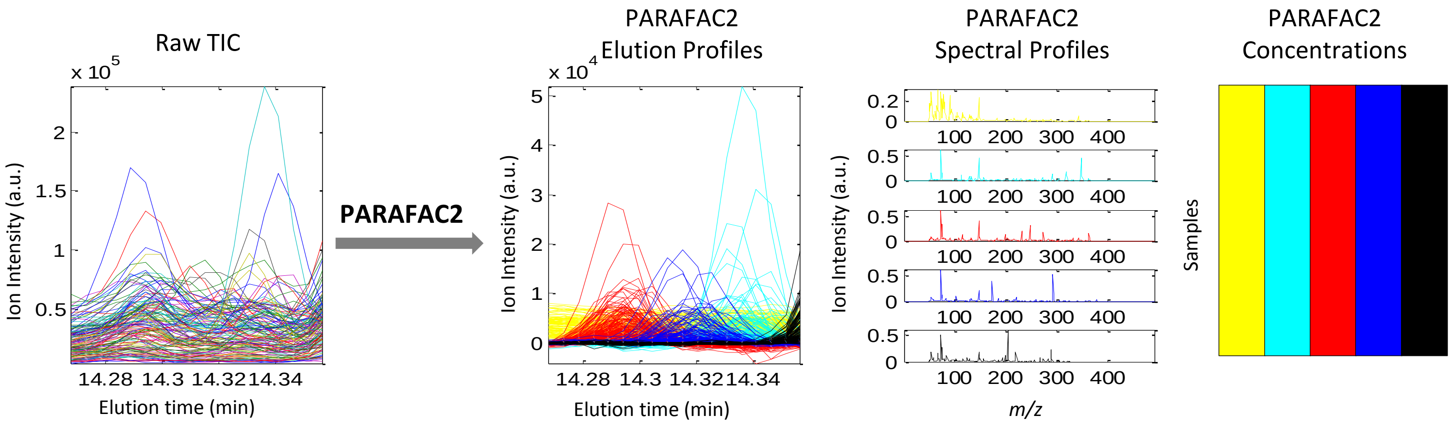

Supplementary Figure S1. Example of raw GC-MS data processing using a multi-way decomposition technique, PARAllel FACtor Analysis 2 (PARAFAC2). This data represents retention time (RT) interval 14.27-14.35 mins and shows deconvolution of 2-Ketoglutaric acid (cyan profile), 3-Hydroxy-3-methylglutaric acid (red profile) and L-Threonic acid (blue profile). Total number of components required was five that explained three above mentioned metabolites, baseline (yellow profile), and the shoulder of the neighbouring peak (black profile) eluting after 2-Ketoglutaric acid (cyan profile). Retention times of deconvoluted peaks (PARAFAC2 elution profiles) were calculated as a mean of RTs of each peak across all samples and based on these RTs, retention indices (RI) of metabolites were estimated using all even alkane mixture sample (C10-C40). PARAFAC2 Spectral Profiles that correspond to EI-MS spectra of metabolites, along with RIs, were used to identify metabolites using NIST11 database. The final metabolite table was constructed using PARAFAC2 concentrations of deconvoluted peaks. After removing components representing baseline, artifact peaks and shoulders of neighbor peaks, 247 deconvoluted peaks with unique RI and EI-MS were obtained from PARAFAC2 models of the raw GC-MS data intervals. Comparison of these peaks against the NIST11 metabolite database identified 104 peaks as being organic acids, aldehydes, alcohols, polyols, phenolic compounds, flavonoids, fatty acids, carbohydrates, and other classes. Nearly 140 peaks remained unknown with unique RI and EI-MS. Detailed information on metabolomics data, identities of metabolites, their parameters including RT, RI, EI-MS similarities are listed in Supplementary Table S1.

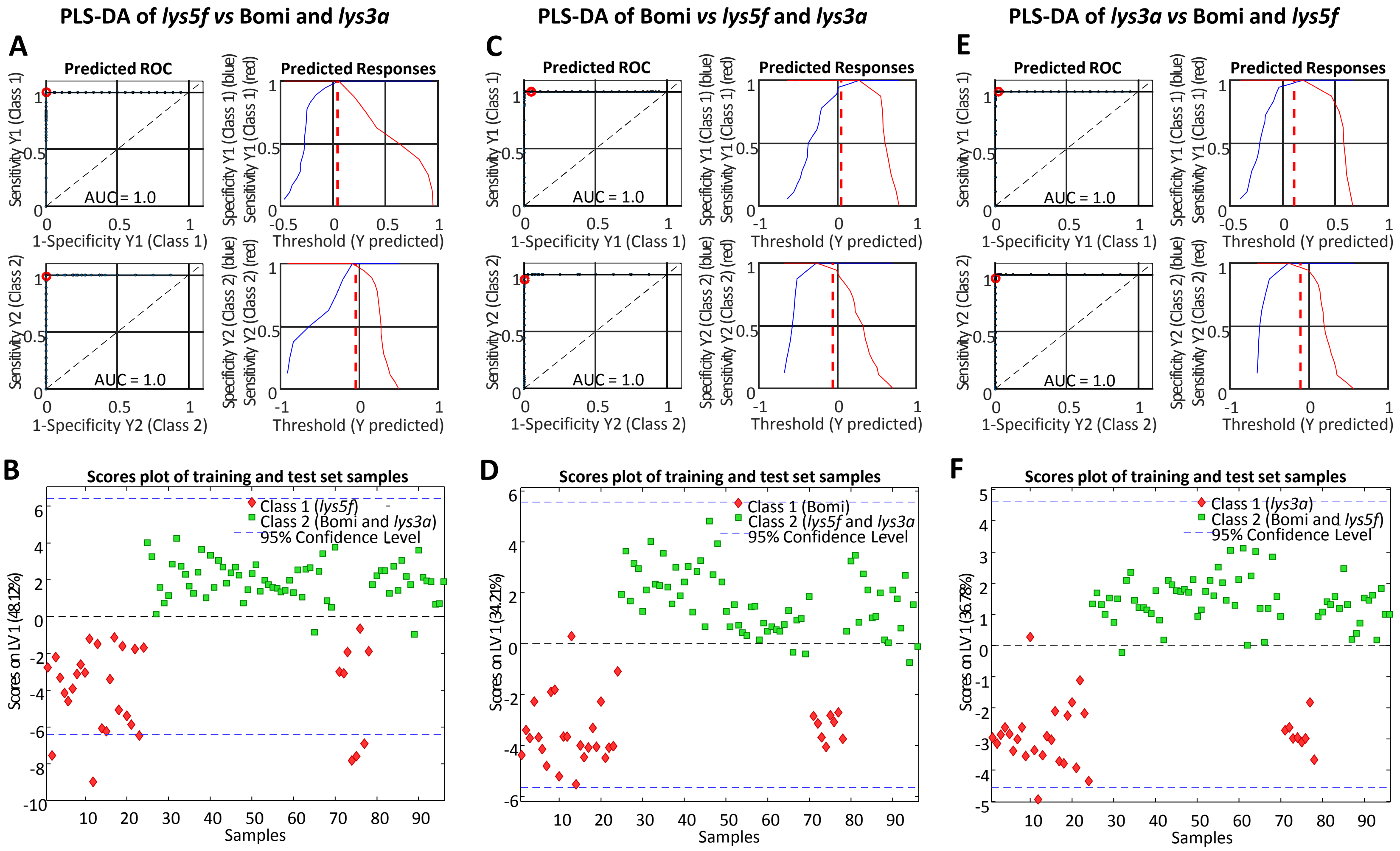

Supplementary Figure S2. Prediction power of the PLS-DA model developed to discriminate barley mutant models. Area Under the Curve (AUC) of Receiver Operating Characteristic (ROC) **(A)** and LV1 scores plot **(B)** of the PLS-DA models developed to discriminate *lys5f* (class 1) from Bomi and *lys3a* (class 2). Panels **(C)** and **(D)**, and **(E)** and **(F)** represent AUC and LV1 scores plot of the PLS-DA models developed to discriminate Bomi (class 1) from *lys5f* and *lys3a* (class 2), and *lys3a* (class 1) from Bomi and *lys5f* (class 2), respectively. All models were optimized using training samples (far left 70 samples in scores plot) and validated using independent test set samples (far right 26 samples in scores plot), as described in the methods section. Most discriminative markers selected using variable selection approach for all three models are depicted in the loadings plot of the PCA model developed on ASCA based genotype effect separated data (Fig. 2B).

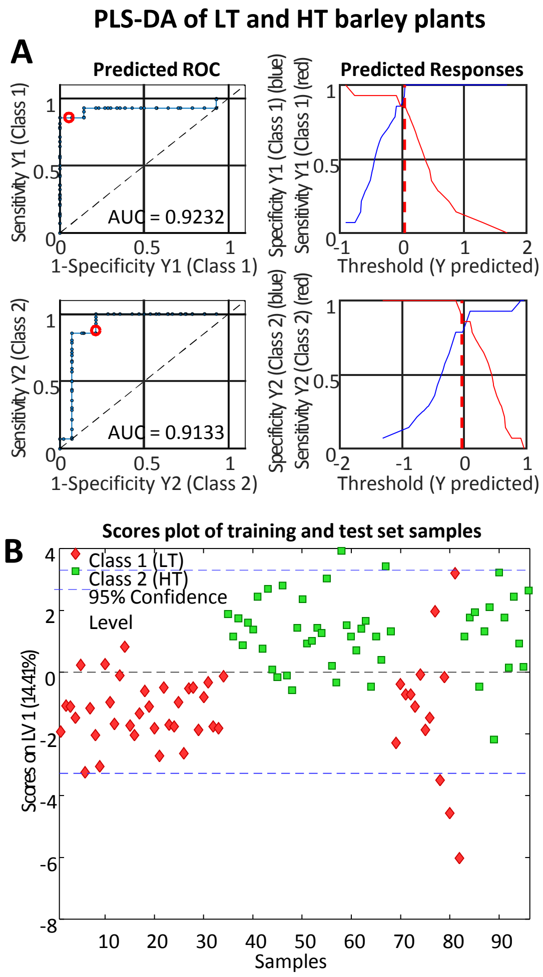

Supplementary Figure S3. Prediction power of the PLS-DA model developed to discriminate barley plants grown under low (LT) and high (HT) temperatures. Area Under the Curve (AUC) of Receiver Operating Characteristic (ROC) **(A)** and LV1 scores plot **(B)** of the PLS-DA model developed to discriminate low temperature (LT), 15 ^{o}C, grown barlye plants from the high temperature (HT), 25 ^{o}C, grown plants. The model was optimized using training samples (far left 70 samples in scores plot) and validated using independent test set samples (far right 26 samples in scores plot), as described in the methods section. Most discriminative markers selected using variable selection approach are depicted in the loadings plot of the PCA model developed on ASCA based growth temperature effect separated data (Fig. 3B).

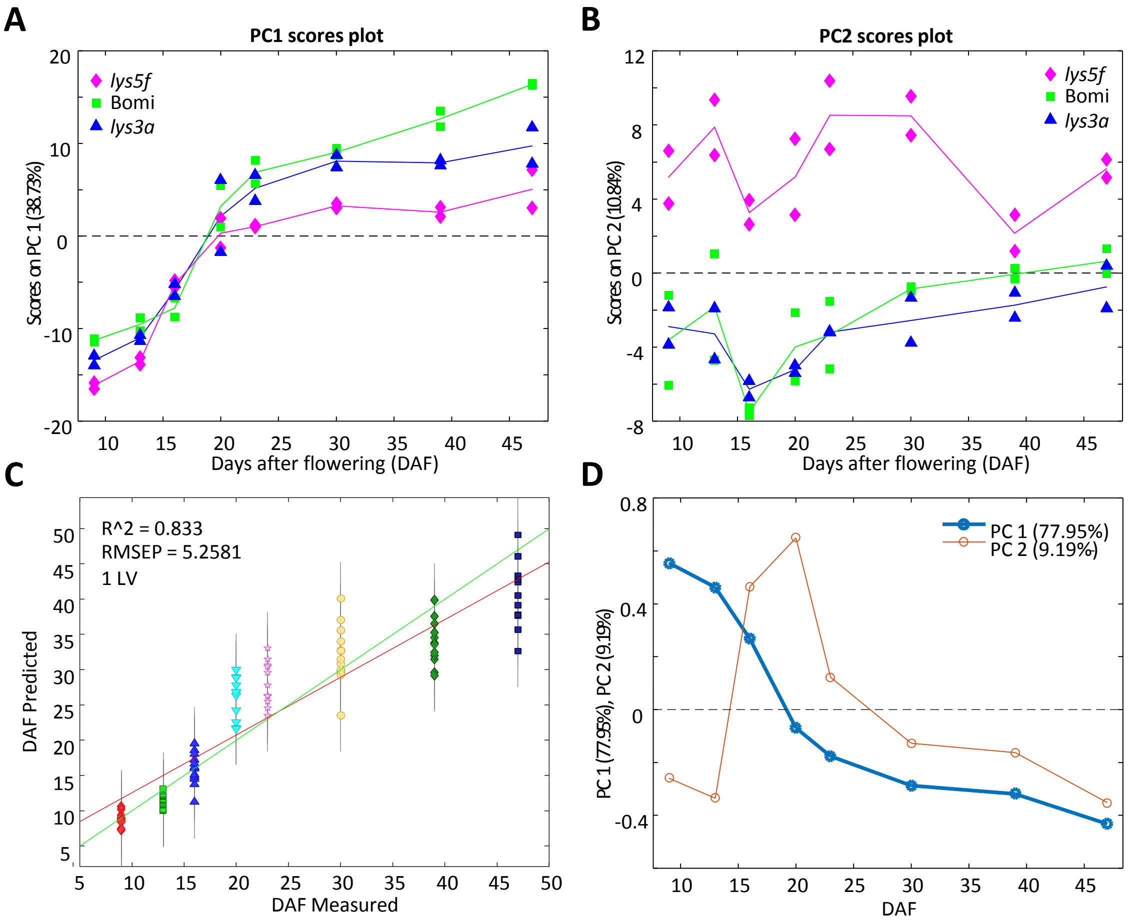

Supplementary Figure S4. The common and GT specific effects of the developmental stages (DAF) on barley seed metabolome. Panels **(A)** and **(B)** show PC1 and PC2 scores plots, respectively, of the PCA model developed on the genotype dependant DAF effect separated data, $\mathbf{X}_{DAF \times GT}$, using ASCA. PC1 over 8 DAF points illustrates metabolite accumulation and depletion trends, however these trend differ among barley genotypes. Dominating trend of accumulation was represented by variables with high loadings on PC1, whereas variables with negative loadings on the PC1 corresponded to the depletion trend. PC2 scores over 8 DAF points depicts rapid increase between 13-20 DAF followed by gradual decrease after 23 DAF. Panel **(C)** illustrates prediction power of the PLS model developed on $\mathbf{X}_{DAF,}$ and Variable Importance for Projection (VIP) scores and regression coefficients of this model allowed identification of the metabolites highly influenced by the DAF. Panel **(D)** shows PC1 and PC2 scores, over 8 DAF points, of the PCA model developed on the $\mathbf{X}_{DAF\_MEAN,}$ data which is the mean of each genotype over 8 DAF points. This PCA model indeed proved that there are only three major trends over which seed metabolome develop during the grain filling stages.

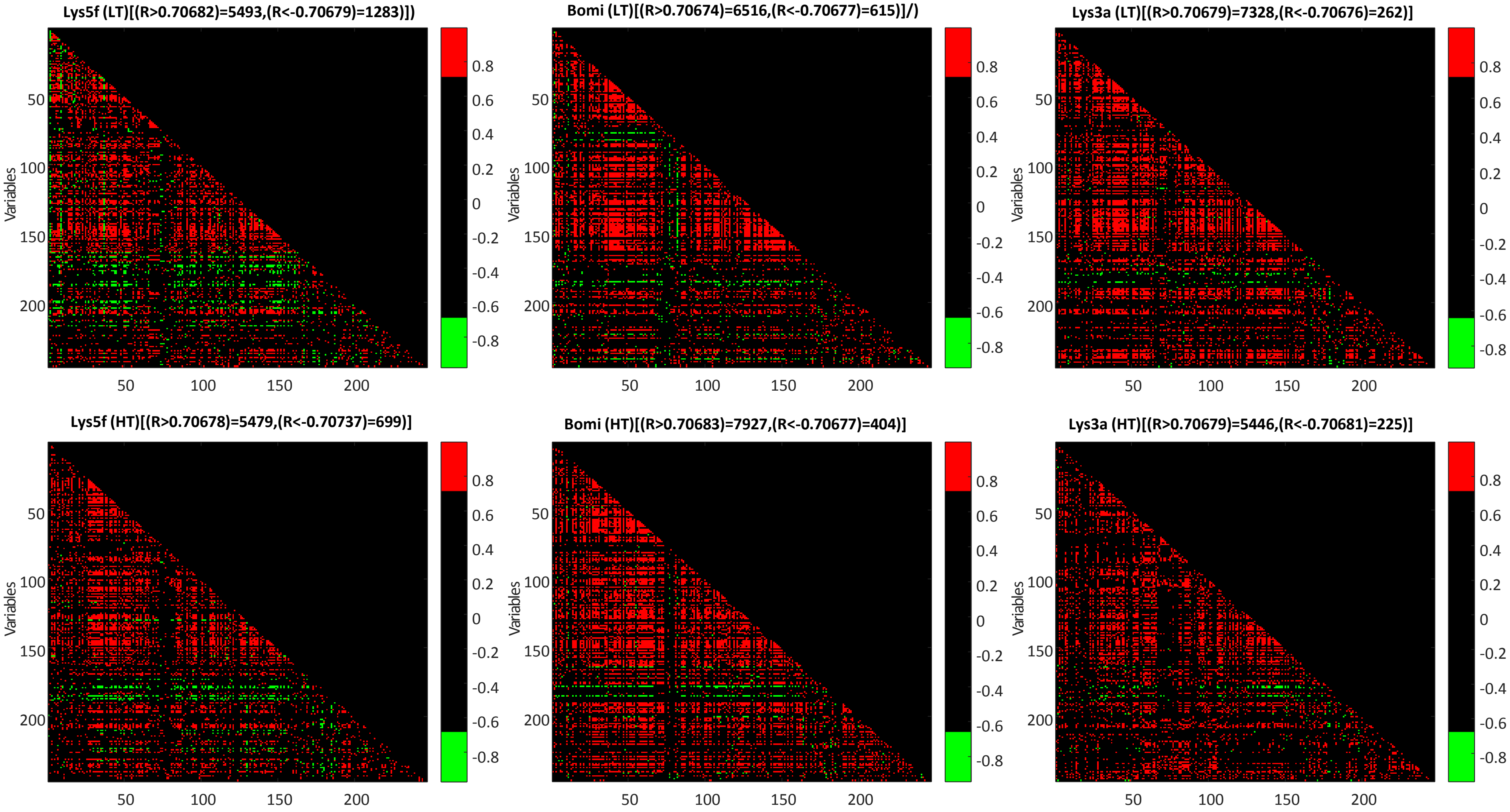

Supplementary Figure S5. Heat maps of Pearson correlation coefficients among metabolites over 8 DAF points within each barley genotype and growth temperature. The $\mathbf{X_{DAF}}$ data was used for each genotype including 8 DAF samples and 247 variables. Heat maps show unique metabolite correlations during the grain filling with differing significantly positive and negative correlations for each genotype. *Headline of each heat map contains information on the number of positive and negative correlations above the above the positive or below the negative significant correlation levels.

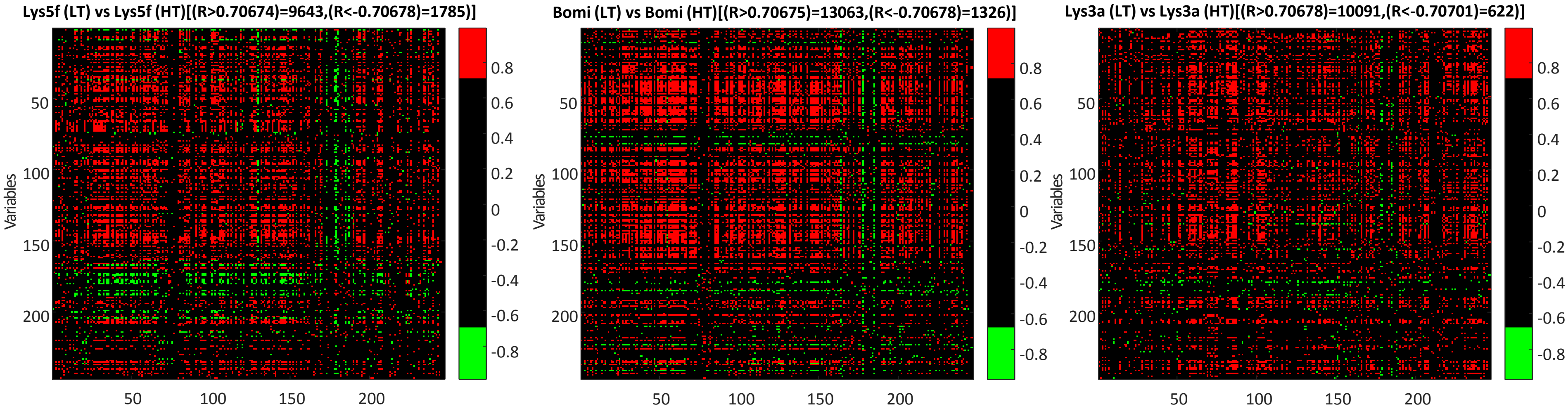

Supplementary Figure S6. Heat maps of Pearson correlation coefficients among metabolites over 8 DAF points between the same barley genotypes growth under low (15 ^{o}C) and high (25 ^{o}C) temperatures. The $\mathbf{X}_{\mathbf{DAF}}$ data was used for each genotype including 8 DAF samples and 247 variables. Heat maps show unique metabolite correlations during the grain filling with differing significantly positive and negative correlations for each genotype. The number of significant positive correlations were notably high in the mother line Bomi compared to mutants. *Headline of each heat map contains information on the number of positive and negative correlations above the above the positive or below the negative significant correlation levels.

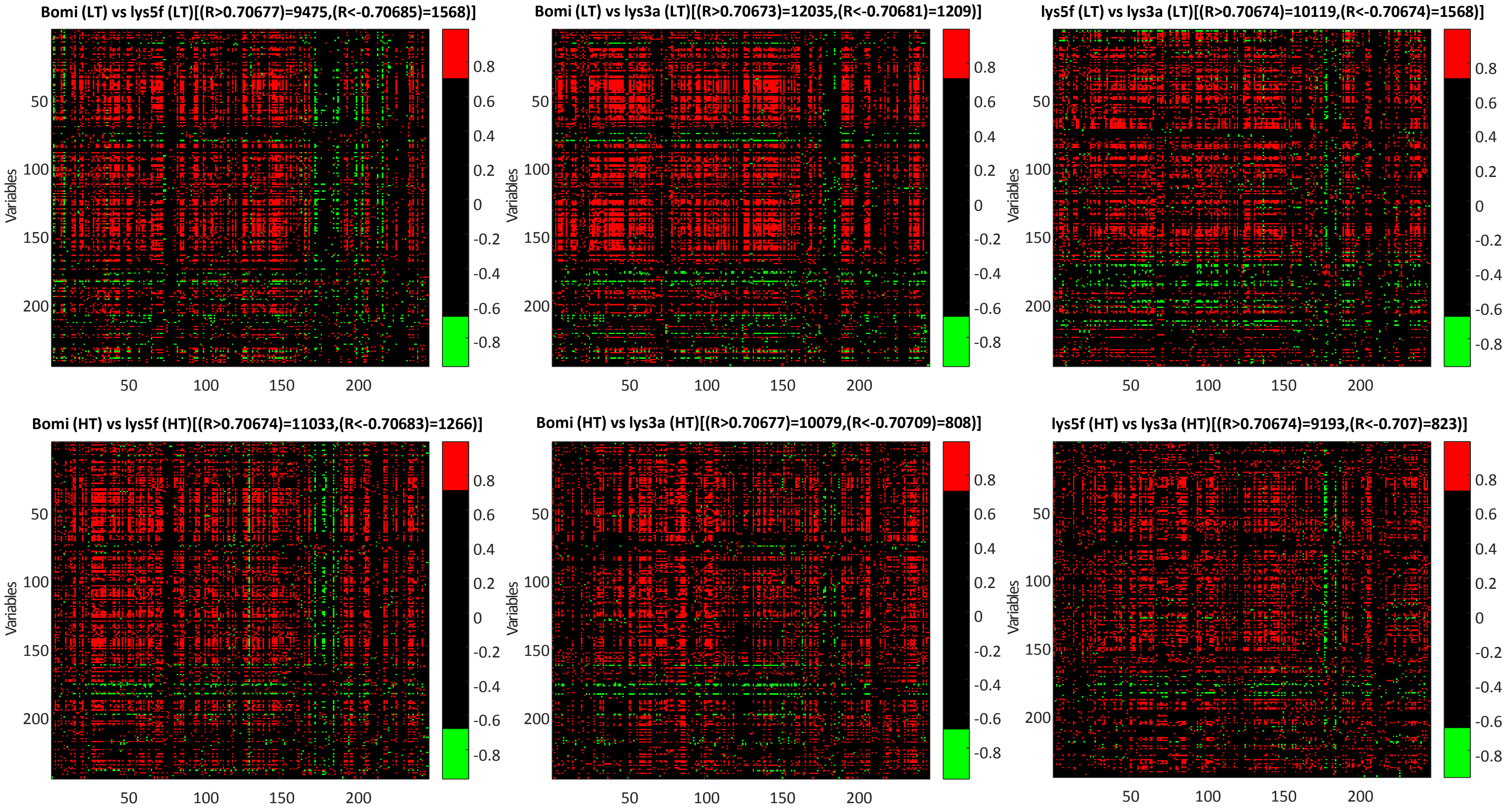

Supplementary Figure S7. Heat maps of Pearson correlation coefficients among metabolites over 8 DAF points between the different barley genotypes growth under the same growth temperatures. The $\mathbf{X}_{\mathbf{DAF}}$ data was used for each genotype including 8 DAF samples and 247 variables. Heat maps show unique metabolite correlations during the grain filling with differing significantly positive and negative correlations for each genotype. *Headline of each heat map contains information on the number of positive and negative correlations above the above the positive or below the negative significant correlation levels.
